# Satisfaction of basic needs mediates relationships between incremental mindsets and well-being

**DOI:** 10.1371/journal.pone.0309079

**Published:** 2024-08-16

**Authors:** Marzena Cypryańska, John B. Nezlek

**Affiliations:** 1 Center for Climate Action and Social Transformations (4CAST), Institute of Psychology, SWPS University, Warsaw, Poland; 2 Department of Psychological Sciences, College of William & Mary, Williamsburg, Virginia, United States of America; University of Tartu, ESTONIA

## Abstract

Research on the extent to which people believe that people can change (incremental beliefs) suggests that incrementalist beliefs are positively related to well-being, whereas entity beliefs (people cannot change) are not. One explanation for this relationship is that incremental beliefs are associated with a mastery orientation, whereas entity beliefs are not. If this is the case, then autonomous and competence motives should mediate relationships between incrementalism and well-being because these motives reflect different aspects of mastery. The present study examined the possibility that autonomous and competence motives mediate relationships between self-theories and well-being. Participants were adult community members (*n* = 428) who completed the Life Engagement Test (eudaimonic well-being), the Satisfaction with life Scale (hedonic well-being), the Mental Health Continuum Scale (eudaimonic, subjective, and psychological well-being), the Basic Needs Satisfaction scale (autonomy, competence, relatedness), and a measure of implicit theories of the self (incremental and entity beliefs). Regression analyses found that incremental beliefs were significantly related (positively) to all three measures of well-being, whereas entity beliefs were not significantly related to well-being. Regression analyses also found that incremental beliefs were positively related to satisfaction of autonomy and competence needs but were not related to satisfaction of relatedness needs. Entity beliefs were not related to the satisfaction of any of the three basic needs. A series of mediational analyses found that competence and autonomy motives mediated relationships between incremental beliefs and all three measures of well-being. In all but one case, satisfaction with life, the direct effects of incremental beliefs on well-being were rendered non-significant when satisfaction of autonomy and competence needs were included as mediators. The present results confirm and extend to the general domain the supposition that a mastery orientation is responsible for relationships between well-being and incremental theories of the self. They also conform the importance of the tenants of Self-Determination Theory in understanding self-theories.

## Introduction

As shown by a considerable body of research, individuals vary in the extent to which they believe that the self can change [1], and Dweck introduced the terms incremental and entity theories to refer respectively to people’s beliefs that the self can change and that it cannot. Research on mindsets, a term that is now commonly used to refer to the distinction between incremental and entity beliefs, has consistently found that incremental beliefs are more adaptive than entity beliefs. For example, performance in school has been found to be positively related to the strength with which students advocate incremental theories of the self, or alternatively, is negatively related to the strength with which students advocate entity theories of the self [e.g., 2]. Moreover, although much of the research on mindsets has concerned achievement (e.g., school performance) mindsets can play roles in various domains [1].

Although numerous underlying mechanisms and processes have been proposed for these relationships [2], few studies, particularly outside of the domain of academic achievement, have examined the essence of Dweck’s contention that “mastery” and “helpless” orientations are responsible (at least in part) for relationships between mindsets and positive outcomes. Regardless, there seems to be broad agreement that relationships between mindsets and various outcomes reflects the fact that incremental beliefs are associated with a mastery orientation, whereas entity beliefs are associated with a helpless orientation.

### Conceptualizing mindsets: One factor or two?

An unresolved issue in the study of mindsets is the extent to which mindsets should be conceptualized as a uni-dimensional construct anchored at one end by incrementalist beliefs and anchored at the other end by entity beliefs, or as two separate constructs. Dweck and colleagues write as if mindsets consist of two components, referring frequently to incrementalist and entitative beliefs as they were distinct rather than opposite ends of a single continuum, but their research consistently represents mindsets with a single score reflecting the extent to which people advocate an incremental mindset.

Nevertheless, as long ago as 1995, Dweck and colleagues acknowledged the possibility that incremental and entity beliefs were distinct constructs [3]. Consistent with this possibility, a meta-analysis of young people’s mindsets regarding intelligence found clear support for two correlated factors [4]. Along the same lines, in a series of studies Karwoski has found that mindsets regarding creativity are best conceptualized as two factors corresponding to incremental and entity beliefs rather than a single factor [e.g., 5]. With this research and the content of the items of the scale in mind, we conceptualized mindsets in terms of two correlated constructs, and we examined how well our data fit this conceptualization before examining relationships among mindsets, well-being, basic need satisfaction, the primary focus of the present study.

### Mindsets and well-being

The present study examined relationships between mindsets and well-being. Typically, the construct of mindsets has been applied to performance of some kind, to success at a task, or to the achievement of a goal. Nevertheless, examination of the items on some measures of mindsets suggests that mindsets can refer to more than achievement or performance [6]. For example, in the mindset questionnaire titled “Kind of person” entity beliefs are measured by items such as “People can do things differently, but the important parts of who they are can’t really be changed.” In contrast, incremental beliefs are measured by items such as “Everyone, no matter who they are, can significantly change their basic characteristics.” Such items refer to life in general, not to a specific domain such as academic achievement or work. As such, we expected that mindsets would be related to well-being defined in general terms.

Contemporary research on well-being suggests that well-being can be defined in terms of hedonic and eudemonic well-being. As noted by Ryan and Deci [7]: “Current research on well-being has been derived from two general perspectives: the hedonic approach, which focuses on happiness and defines well-being in terms of pleasure attainment and pain avoidance; and the eudaimonic approach, which focuses on meaning and self-realization and defines well-being in terms of the degree to which a person is fully functioning.” Although Deci and Ryan discuss how hedonic and eudemonic approaches differ and how they overlap, in terms of mindsets, we believe that possessing an incremental mindset should be associated with greater well-being whether well-being is conceptualized in hedonic or eudemonic terms. The sense of mastery inherent in an incremental mindset should facilitate both types of well-being, a supposition consistent with self-determination theory [SDT; 8] and with research about well-being that has been conducted within the context of SDT.

### Self-determination theory and satisfaction of basic needs

Self-determination theory is a major theoretical framework that concerns human motivation broadly defined [9, 10]. We choose SDT as our explanatory framework because of its breadth and its emphasis on constructs that we believed corresponded to the mastery orientation discussed by Dweck and colleagues. SDT. SDT discusses human motivation in terms of three needs: competence, autonomy, and relatedness. Within the context of SDT, we assumed that competence and autonomy corresponded to mastery as discussed by Dweck and colleagues.

### Satisfaction of basic needs as mediators of relationships between mindsets and well-being

The extent to which the basic needs proposed by SDT are satisfied has been found to be positively related to well-being across various domains and cultures [11]. This suggested to us that need satisfaction might mediate relationships between mindsets and well-being. Dweck and colleagues have proposed that a sense of mastery is responsible for relationships between mindsets and well-being. We collected data that allowed us to determine if the extent to which the satisfaction of the basic needs of competence and autonomy mediated relationships between mindsets and well-being. We chose the satisfaction of the basic needs for competence and for autonomy because we thought that people’s beliefs about their competence in terms of life activities and their beliefs about the extent to which they were free to choose what they did (autonomy) were aspects of mastery.

Although the construct of “need satisfaction” may not immediately lead to an association with feeling autonomous or competent for some, inspection of the items on the Basic Needs Satisfaction Scale [BNS; 12] scale suggests that need satisfaction overlaps considerably with people’s beliefs about how competent or autonomous they are. For example, two items on the competence subscale of Gagne’s measure are: “Most days I feel a sense of accomplishment from what I do,” and “Often, I do not feel very competent” (reverse-coded). Two items on the autonomy scale are: “I generally feel free to express my ideas and opinions,” and “There is not much opportunity for me to decide for myself how to do things in my daily life” (reverse-coded). The scales measure how competent and autonomous a person feels, which is a measure of how well these needs are satisfied.

We understood mediation as described by Agler and De Boeck [13]: ‘…mediation processes are framed in terms of intermediate variables between an independent variable and a dependent variable, with a minimum of three variables required in total: X, M, and Y, where X is the independent variable (IV), Y is the dependent variable (DV), and M is the (hypothesized) mediator variable that is supposed to transmit the causal effect of X to Y. The total effect of X on Y is referred to as the total effect (TE), and that effect is then partitioned into a combination of a direct effect (DE) of X on Y, and an indirect effect (IE) of X on Y that is transmitted through M. In other words, the relationship between X and Y is decomposed into a direct link and an indirect link.”

In their classic description of mediation Baron and Kenny [14] described three criteria for mediation. (1) The predictor needs to be related to the outcome, or there is nothing to mediate, although there may be exceptions to this rule such as suppression [13, 15]. (2) The predictor needs to be related to the mediator; otherwise, the mediator cannot mediate the relationship between the predictor and the outcome. (3) Including the mediator should lead to a reduction in the strength of the relationship between the predictor and the outcome. This is a simplified description of mediation. See Hayes [16] for a more thorough discussion of mediation.

### Research questions and expectations

Within the present context and in terms of the mediational model to be tested, well-being is the outcome, mindsets are the predictors, and the satisfaction of basic needs are the mediators. We proposed this model assuming that mindsets lead to (or precede) the satisfaction of basic needs, and that in turn, the satisfaction of basic needs leads to (or precedes) well-being. One of the tenets of mindset theory is that incremental mindsets are associated with a sense of mastery over one’s environment (or some specific domain of life). In terms of SDT and BNS, mastery is represented by autonomy, a belief that one is acting on his/her own volition, and competence, a belief that one can achieve goals and accomplish tasks.

Therefore, we expected that the satisfaction of autonomy and competence needs (i.e., how autonomous and competent a person feels) would mediate relationships between incremental mindsets and well-being. Given that we distinguished incremental and entity mindsets, we assumed that this mediation would exist only for incremental mindsets, not for entity mindsets. Finally, mediation, particularly within the context of a cross-sectional design with only one measurement occasion, does not provide a strong basis for drawing causal inferences, and we discuss the issue of causal precedence in the discussion section.

## Method

### Participants

Participants were 428 adults (*M*_age_ = 33.0 years, *SD* = 7.84, range 18–59 years; 211 women) who were recruited via calls on the internet to participate in a study on running and well-being. Data were collected from two samples, which were combined for the present analyses. The first sample was obtained between 21 March and 26 May 2015, and the second between 23 April and 22 August 2016. For both samples, participants were told that their names would be entered into a lottery for prizes ranging from running shoes and other running paraphernalia to an all-expense paid trip to a run held in Europe, and these prizes were awarded at the conclusion of each study.

### Ethical statement

The study was conducted in accordance with the Declaration of Helsinki regarding the rights of research participants. Participants consented electronically by clicking on a link indicating their agreement to participate after being told that their names would not be associated with their answers and that they could terminate participation at any time without penalty. Consistent with these instructions, responses were de-identified prior to analysis. Ethics approval was obtained from the Ethics Committee for Scientific Research Involving Humans as subjects, School of Social Psychology, Campus in Poznań, protocol 5/2015/WZ, entitled "Personality traits, specific patterns of adaptation and well-being and related activity with preparation for long-distance runs (National study runners’ motivation),” approved on 18 March 2015.

### Measures

#### Mindsets

Mindsets were measured with the eight-item “Kind of person” scale discussed by Dweck [6]. The scale has four items that measure incrementalist beliefs, e.g., “People can always substantially change the kind of person they are,” and four items that measure entity beliefs, e.g., “Everyone is a certain kind of person, and there is not much that can be done to really change that.” We used a Polish language version of the scale developed by Lachowicz-Tabaczek [17]. Responses were made using a six-point scale with endpoints labeled: 1 = definitely disagree and 6 = definitely agree.

#### Satisfaction of basic needs

Satisfaction of basic needs was measured with the Basic Psychological Needs Satisfaction Scale [BNS; 8, 12]. The BNS consists of 21 items, seven items for each of the three subscales: Autonomy, Competence, and Relatedness. To create a Polish language version of the scale, the items on the scale were translated and back-translated by a team consisting of members fluent in both languages, some of whom had 25+ years of experience. Participants responded using seven-point scales with endpoints labeled: 1 = not at all true and 7 = completely true.

#### Well-being

Well-being was measured with three scales: the Mental Health Continuum Short Form [MHC; 18], the Life Engagement Test [LET; 19], and the Satisfaction with Life Scale [SWLS; 20]. We used a Polish language version of the MHC created by Karaś et al. [21]. The MHC has three subscales: Eudaimonic, Social, and Psychological. Satisfaction with life was measured using a Polish language version of the SWLS created by Jankowski [22], and Life Engagement was measured using a Polish language version of the LET created by Bąk et al. [Unpublished]. Due to a programming error, item three of the MHC was not administered.

Responses to the MHC were made using six-point scales with endpoint labeled: 0 = ‘never’ and 5 = ‘every day.’ Responses to the LET were made using five-point scales with endpoint labeled: 1 = definitely disagree and 5 = definitely agree. Responses to the SWLS were made using seven-point scales with endpoints labeled: 1 = definitely disagree and 7 = definitely agree.

## Results

### Factor structure of the measure of mindsets

Before conducting the primary analyses of the study, we examined the factor structure of the measure of mindsets. Based upon an inspection of the items and previous research [23, e.g., 24], we used Mplus to conduct a confirmatory factor analysis that examined the fit between the data and a two factor model. We modeled items 1, 4, 7, and 8 as observed measures of a latent construct of incrementalist beliefs, and items 2, 3, 5, and 6 as observed measures of a latent construct of entity beliefs. The covariance between the factors was left to vary freely.

This analysis found that the proposed two-factor model fit the data well (CFI = .978; TFI = .978, SRMR = .029, RMSEA = .074, 95% CI = .055/.094), and the standardized coefficients are presented in Table 1. The estimated standardized covariance between the two factors was -.776. The results of this analysis are summarized in Table 1.

**Table 1:** Results of confirmatory factor analysis for mindsets measure.

Coefficients		
Incremental	Entity	Item
.713		Everyone, regardless of their nature, can significantly change their character traits
.588		Whether people can change depends only on their determination
.886		No matter what kind of person someone is, they can always change very much
.917		Everyone can change their even the most basic character traits.
	.753	The person you are is very basic and cannot be changed
	.878	People cannot significantly change their basic character traits.
	.830	Each person has his own personality and even if he tried, he will not be able to change it
	.716	People can do things differently, but who they are can’t really be changed.

We also fit a single factor model, but this model did not appear to fit the data as well as the two-factor model. Although it was not possible to compare statistically the fits of these two models because they had the same *df*, for all fit indices, the fit of the two-factor model was better. For example, e.g., the sample-size adjusted BIC was 8575.4 for the two-factor model, and it was 8660.2 for the single factor model. The χ2 difference between the models was 84.8, and for the one factor model the TFI was .90 and the RMESA was .13.

Based on these analyses, we computed separate scores representing incremental and entity beliefs. These were defined as the mean of the four items that measured incremental beliefs (items 1, 4, 7, and 8) and the mean of the four items that measured entity beliefs (items 2, 3, 5, and 6).

### Descriptive statistics

Before conducting the primary analyses of the study, we examined the means, standard deviations, reliabilities, and correlations between our measures. These summary statistics are presented in Table 2. According to guidelines provided by Shrout [25], all measures had at least moderate reliability (.61 to .80), and most had substantial reliability (.81 and above).

**Table 2:** Descriptive statistics, reliabilities, and correlations for measures.

	*M*	*SD*	α	Entity	Autonomy	Competence	Relatedness	SWLS	LET	MHC-E	MHC-S	MHC-P
Incremental	4.30	.90	.86	-.678	.297	.285	.197	.287	.287	.231	.186	.261
Entity	2.87	.97	.87		-.228	-.202	-.143	-.150	-.197	-.132	-.072	-.149
Autonomy	4.99	.87	.79			.677	.582	.572	.630	.531	.467	.646
Competence	4.88	.85	.75				.570	.583	.666	.559	.521	.673
Relatedness	5.35	.78	.81					.473	.514	.429	.471	.532
SWLS	4.50	1.10	.89						.620	.611	.479	.632
LET	4.06	.61	.78							.581	.485	.633
MHC-E	3.25	1.06	.82								.617	.724
MHC-S	2.44	1.13	.87									.726
MHC-P	3.26	1.02	.90									

Note: MHC-E, Mental Health Continuum Eudaimonic well-being; MHC-S, Mental Health Continuum Social well-being

MHC-P, Mental Health Continuum Psychological well-being. All coefficients were significant at *p* < .05; |r| > .125, *p* < .01.

#### Relationships between mindsets and well-being

The first requirement for mediation is that a predictor is related to an outcome; otherwise, there is nothing to mediate. To determine if our data met this requirement, we examined relationships between the measures of the two mindsets (predictors) and well-being (outcomes) with a set of regression analyses in which incrementalist beliefs and entity beliefs were regressed onto each measure of well-being. The results of these analyses were quite clear, and the results are summarized in Table 3. When incremental and entity beliefs were considered simultaneously, incremental beliefs were significantly and positively related to all measures of well-being, whereas entity beliefs were not significantly related to any measure of well-being.

**Table 3:** Summary of analyses of mindsets regressed onto well-being.

			Incremental beliefs		Entity beliefs	
Measure	*F*-ratio	*R* ^2^	β	*t*-ratio	β	*t*-ratio
MHC-E	12.17***	.054	.260	4.06***	.044	< 1
MHC-S	8.83***	.040	.253	3.92***	.099	1.53
MHC-P	15.80***	.070	.295	4.63***	.050	< 1
LET	19.06***	.082	.284	4.49***	-.004	< 1
SWLS	20.05***	.086	.344	5.45***	.083	1.32

Note: All F-ratios were significant at *p* < .001. All coefficients for Incremental beliefs were significant at *p* < .001. No coefficients for Entity beliefs were significant at *p* < .05.

**Relationships between mindsets and satisfaction of basic needs.** The second requirement for mediation is that predictors are related to potential mediators. To determine if our data met this requirement, we examined relationships between the measures of the two mindsets and satisfaction of basic needs in regression analyses in which incremental beliefs and entity beliefs were regressed onto each measure of satisfaction of basic needs. The results of these analyses were also quite clear, and the results are summarized in Table 4. When incremental and entity beliefs were considered simultaneously, incremental beliefs were significantly and positively related to all three measures of satisfaction of basic needs, whereas entity beliefs were not significantly related to any measure of the satisfaction of basic needs.

**Table 4:** Summary of analyses of mindsets regressed onto satisfaction of basic needs.

			Incremental		Entity	
Measure	*F*-ratio	*R* ^2^	β	*t*-ratio	β	*t*-ratio
Autonomy	20.97***	.090	.264	4.19***	.049	< 1
Competence	18.88***	.082	.275	4.35***	-.015	< 1
Relatedness	8.65***	.039	.186	2.88***	-.16	< 1

See note for Table 3.

#### Relationships between satisfaction of basic needs and well-being

Although relationships between mediators (satisfaction of basic needs) and outcomes (well-being) do not figure prominently in discussions of mediation, we examined these relationships nevertheless. This reflected in part our desire to have a complete understanding of the relationships among the variables we measured. This is consistent with what Agler and De Boeck [13] described as an “indirectness perspective” (p. 4). In addition, the results of these analyses contribute to our understanding of relationships between well-being and the satisfaction of basic needs.

Relationships between satisfaction of basic needs and well-being were examined in a series of regression analyses in which the three measures of satisfaction of basic needs were regressed onto each measure of well-being. The results of these analyses are summarized in Table 5. As can be seen from these results, with only one exception, the satisfaction of all three basic needs was significantly and positively related to all measures of well-being. The exception was the relationship between the satisfaction of relatedness needs and MHC Eudemonia, which was significant at *p* = .07. Such relationships are consistent with the contention of SDT that the satisfaction of basic needs provides a foundation for well-being.

**Table 5:** Summary of analyses of satisfaction of basic needs regressed onto measures of well-being.

			Autonomy		Competence		Relatedness	
Measure	*F*-ratio	*R* ^2^	β	*t*-ratio	β	*t*-ratio	β	*t*-ratio
MHC-E	79.05***	.360	.247	4.41***	.339	6.11***	.092	1.83^a^
MHC-S	67.50***	.324	.130	2.25*	.307	5.39***	.220	4.28***
MHC-P	158.58***	.530	.303	6.30***	.393	8.27***	.131	3.05**
LET	147.64***	.511	.289	5.92***	.406	8.40***	.114	2.62**
SWLS	97.31***	.408	.281	5.23***	.321	6.05***	.126	2.62**

Note: *** *p* < .001

** *p* ≤ .01

* *p* ≤ .05

^a^ *p* = .07

#### Basic needs as mediators of relationships between incremental beliefs and well-being

The preceding analyses were conducted in anticipation of the mediational analyses, which are the primary focus of this paper. After controlling for the relationship between incremental and entity mindsets, entity mindsets were not related to any measure of well-being, and entity mindsets were not related to any measure of satisfaction of basic needs. In other words, the measure of entity mindsets did not meet either criterion for mediation.

Given this, the mediational analyses were limited to how the satisfaction of basic needs mediated relationships between incremental mindsets and well-being. The present hypotheses concern how mastery mediates relationships between incremental mindsets and well-being, and only two of the three basic needs, autonomy and competence, concern mastery. Therefore, the mediational analyses focused on satisfaction of competence and autonomy needs as mediators.

The mediational analyses were done using the PROCESS macro [16], Model 4. In these analyses, a measure of well-being was the outcome, a score representing incremental beliefs was the predictor, and the satisfaction of autonomy and competence needs were the mediators. Both mediators were included simultaneously. To obtain 95% confidence intervals (CI), 5,000 bootstrap iterations were made. The results of these analyses are summarized in Table 6. We present unstandardized coefficients to provide a basis to understand how total effects were partitioned into direct and indirect effects. To interpret the statistical significance of effects, we treated effects for which the CIs did not include 0 as different from 0.

**Table 6:** Summary of mediation analyses.

Measure	total effect	direct effect	total indirect	% indirect	Autonomy	Competence
MHC-E	.276 (.165/.387)	.053 (-.044/.150)	.223 (.154/.293)	80.8%	.097 (.051/.152)	.126 (.071/.188)
MHC-S	.236 (.117/.355)	.019 (-.089/.126)	.217 (.148/.290)	91.9%	.078 (.030/.135)	.139 (.082/.205)
MHC-P	.300 (.194/.406)	.038 (-.043/.118)	.262 (.185/.344)	87.3%	.118 (.073/.172)	.144 (.090/.205)
LET	.197 (.134/.259)	.048 (-.001/.096)	.149 (.105/.197)	75.6%	.065 (.038/.097)	.084 (.052/.123)
SWLS	.351 (.240/.463)	.118 (.023/.213)	.233 (.160/.308)	66.4%	.112 (.065/.170)	.121 (.070/.176)

The results of these analyses were quite clear. The satisfaction of both autonomy and competence needs mediated the relationship between incremental beliefs and well-being for all five measures of well-being. Moreover, for the three MHC measures and the LET, the CI for the direct effect included 0, i.e., the direct effects were not different from 0. Also, tests of the differences of the indirect effects for autonomy and competence did not find any significant differences between them.

Although there is a robust debate about how to assess the strength of indirect effects in mediation, the indirect effects in the present analyses represented two-thirds or more (some close to 90%) of the total effects. This, combined with the lack of significant direct effects, provides strong support for the central hypothesis of the study that individual differences in mastery are responsible for relationships between incremental mindsets and well-being.

## Discussion

The results clearly supported our hypothesis that a sense of mastery mediates relationships between incremental mindsets and well-being. We found that the satisfaction of the basic needs of Autonomy and Competence mediated relationships between incremental beliefs and well-being for all measures of well-being. Moreover, this mediation was strong. For all but one measure of well-being, the direct effect between incremental beliefs and well-being was not significant after Autonomy and Competence were included as mediators. Some refer to this as “full mediation.” These results support our logic that the sense of mastery that has been discussed as one of the important reasons incrementalist beliefs are associated with success also applies to relationships between incrementalist beliefs and well-being.

The present results meaningfully extend the domain in which mindsets operate. Much of the work on mindsets has concerned performance in achievement domains such as school and work. The present results suggest that people’s beliefs about how they can changeare related to their well-being, generally defined. Such a possibility complements the extensive body of research demonstrating that perceived control over one’s environment is positively related to well-being

### Entity beliefs as distinct from incremental beliefs

Although mindsets were initially conceptualized as an “either/or” single continuum that reflects a single construct, the present results suggest that it may be useful to conceptualize mindsets in terms of two, albeit related constructs: incremental mindsets and entity mindsets. Support for this contention comes from two sources: the results of confirmatory factor analyses and the results of analyses of relationships between mindsets and well-being. The CFA found solid support for the existence of two separate constructs, constructs that made conceptual sense.

The validity of conceptualizing mindsets as two constructs was demonstrated by the differences in the relationships between these two constructs and well-being. At the zero-order, incremental beliefs were positively correlated with well-being, whereas entity beliefs were negatively correlated with well-being. Moreover, when the two measures of mindsets were simultaneously regressed onto measures of well-being, entity beliefs were not significantly related to any measure of well-being, whereas incremental beliefs were positively related to all measures of well-being. This indicates that relationships between well-being and entity beliefs reflect the relationship between entity and incremental beliefs.

Moreover, the possibility that mindsets can be conceptualized in terms of two separate (albeit correlated) dimensions was discussed by Dweck et al. [3]. In responding to numerous commentators in an issue of *Psychological Inquiry*, Dweck et al. (pp. 323–324) noted:

“For simplicity’s sake, we have tended to portray the two implicit theories as mutually exclusive alternatives…Nonetheless, students of the human mind know that the fact that two beliefs are opposites does not prevent people from holding them both…This possibility-that many people actually hold both theories, albeit to differing degrees- raises many other intriguing possibilities and suggests that research into the circumstances that might elicit the different theories may well be in a fruitful direction.”

Despite this response about intriguing possibilities and fruitful directions, nearly 30 years later, the issue of the dimensionality of mindsets has not been explored very vigorously.

Regardless, conceptualizing mindsets as being comprised of two separate, but related dimensions is similar to distinctions that have been made in other domains. For example, in their seminal article, Cacioppo and Berntson [26] discussed how negative and positive evaluations are best conceptualized as a two-dimensional evaluative space, not single, bi-polar dimension. Along the same lines, Elliot and Thrash [27] demonstrated that approach and avoidance motives are best conceptualized as two separate motives, not opposite ends of a single continuum. Earlier, Diener and Emmons [28] found that reports of positive and negative affect were uncorrelated over longer periods of times (e.g., a year), and even though they were negatively correlated over shorter periods of time, these correlations were not strong enough to support a uni-dimensional or bi-polar model.

### Are entity mindsets negatively related to well-being?

Although individual differences in entity mindsets were not related to well-being or to the satisfaction of basic needs when entity mindsets were analyzed with incremental mindsets, they were significantly (negatively) related to both well-being and to the satisfaction of basic needs at the zero-order (simple correlations). These correlations suggest that entity mindsets are not adaptive. Such correlations may represent the existence of what is sometimes referred to as a “pessimistic explanatory style” that has been found to interfere with people’s ability to cope with stress, and more generally, to thrive [29]. It is possible that entity mindsets are, at least in part, a manifestation of the same beliefs, cognitions, and emotions that characterize a pessimistic explanatory style and related constructs.

Nevertheless, the present results are somewhat ambiguous about such possibilities. That is, entity mindset did not play a role, even a minor role, in relationships among mindsets, the satisfaction of basic needs, and well-being when incremental mindsets were included in the analyses. The present results suggest that whatever relationships exist between entity mindsets and well-being or the satisfaction of basic needs are subsumed by relationships between incremental mindsets and these other constructs. Given the lack of attention to the possibility the mindsets may be best conceptualized as two factors rather than one, there is little research or theory that can be used to provide a context for the present results. This will require future research that focuses on this possibility.

### Causal precedence

Within the context of a cross-sectional, single-occasion design such as that used in the present study, demonstrating that a variable (M) mediates a relationship between an outcome (Y) and a predictor (X) cannot serve as a basis for claims about causal relationships among the constructs being measured. Nevertheless, the fact that M mediates a relationship between X and Y can be interpreted as being consistent with, or supportive of, the existence of a causal sequence from X to M to Y. Such support can be meaningful when a mediational model is theory-driven, and when the components of the model represent relationships that have been supported by past research.

We think the present results provide credible support for the existence of a causal sequence from incremental beliefs through the satisfaction of autonomy and competence basic needs to well-being. The direct relationship between incremental beliefs and well-being is a straightforward extension of research on relationships between incremental beliefs and success in other domains. Dweck and colleagues have repeatedly discussed how incremental beliefs are associated with a mastery orientation, and it is this mastery orientation that is responsible for the relationship between incremental beliefs and success. The satisfaction of the basic needs of autonomy and competence are clearly components of a mastery orientation, and so the fact that they mediate relationships between incremental beliefs and well-being is fully consistent with Dweck’s model and the considerable body of research that has been conducted to examine it.

Nonetheless, as just described, the existence of mediation can be used to support, but not prove causality. Causality is best examined using experimental methods, although attempts to manipulate mindsets have produced mixed results [e.g., 30]. Other ways of examining causality include longitudinal models in which changes in mindsets and well-being are measured across time.

### Conclusions

The present results suggest that: (1) mindsets can be conceptualized in terms of two independent sets of beliefs, incremental and entity, (2) incremental beliefs are positively related to well-being defined broadly, and (3) relationships between incremental beliefs and well-being reflect underlying relationship between incremental beliefs and mastery orientation and between a mastery orientation and well-being. Each of these findings is new, and collectively, they provide a basis for new directions in research on mindsets.
